# A novel tumor suppressor SPINK5 targets Wnt/β‐catenin signaling pathway in esophageal cancer

**DOI:** 10.1002/cam4.2078

**Published:** 2019-03-13

**Authors:** Qian Wang, Qin Lv, Hua Bian, Lei Yang, Ke‐lei Guo, Song‐shan Ye, Xue‐feng Dong, Ling‐Ling Tao

**Affiliations:** ^1^ Zhang Zhongjing College of Chinese Medicine Nanyang Institute of Technology Nanyang China; ^2^ Henan Key Laboratory of Zhang Zhongjing Formulae and Herbs for Immunoregulation Nanyang Institute of Technology Nanyang China; ^3^ Nanyang Medical College Nanyang China

**Keywords:** cell migration, cell proliferation, esophageal cancer, GSK3β phosphorylation, *SPINK5*, Wnt/β‐catenin signaling pathway

## Abstract

Esophageal cancer is one of the most common tumor in the world, and the morbidity rate is as high as 100/100 000 in some parts of China. Therefore, it is important and urgent to explore the pathogenesis of esophageal cancer and find new therapeutic targets for esophageal cancer. In this study, we found that a novel tumor suppressor *SPINK5* is significantly reduced in the development of esophageal cancer, and is closely related to the pathological differentiation and lymph node metastasis of esophageal cancer via bioinformatics analysis and esophageal cancer tissue array. Further studies have found that *SPINK5* is closely related to Wnt/β‐catenin signaling pathway by bioinformatics analysis and western blot. In esophageal cancer cells, *SPINK5* overexpression can inhibit Wnt/β‐catenin signaling pathway. Combined with LiCl or MG‐132 treatment, *SPINK5* can inhibit GSK3β phosphorylation and promote β‐catenin protein degradation, thus inhibit Wnt/β‐catenin signaling pathway. In vivo study, *SPINK5 *overexpression can significantly inhibit the growth of esophageal cancer cells. Our study shows that *SPINK5* can inhibit the proliferation, migration, and invasion of esophageal cancer cells by inhibiting Wnt/β‐catenin signaling pathway, and thus plays an important role in the development of esophageal cancer, and may serve as a treatment target of esophageal cancer.

## 1 INTRODUCTION

According to Global cancer statistics 2018, esophageal cancer is the ninth most common tumor in the world. In 2018, there were 572 034 new cases of esophageal cancer. In addition, there were 508 585 (5.3%) patients with esophageal cancer died, which is the sixth leading cause of cancer death worldwide.^1^ More than 70% of esophageal cancer cases worldwide occurred in China, with esophageal squamous cell carcinoma accounting for more than 95%.^2^ In China, despite the continuous improvement of esophageal cancer diagnosis and treatment measures, the prognosis of patients with esophageal cancer is still poor, and the 5‐year survival rate ranges from 10% to 25%.^3^ In addition, in some parts of China, the morbidity rate is as high as 100/100 000, which has become a world‐recognized area with high incidence of esophageal cancer. In these areas, esophageal cancer has become the second largest cancer killer.^4^ Therefore, it is an urgent research topic to continue to explore the pathogenesis of esophageal cancer and to find potential therapeutic targets for esophageal cancer.

The serine protease inhibitor kazal type (*SPINK*) family is the largest branch of the serine protease inhibitor family.^5^ Its family members include *SPINK1*, *SPINK2*, *SPINK*4, *SPINK*5, *SPINK5L2*, *SPINK5L3*,* SPINK6*, *SPINK7*, and *SPINK9*.^6^ Later, *SPINK 8*, *SPINK 10*,* SPINK11*, and *SPINK12* were found in the rat’s epididymis.^7^ The *SPINK *family consists of the Kazal domain. A typical Kazal domain contains 3 pairs of disulfide bonds, 2 a‐helices, and 3 anti‐parallel β‐sheets.^8^ The *SPINK5* gene is located in the 5q32 region of the chromosome and is composed of 15 functional regions. The *SPINK5 *gene encodes a lymphoid epithelial‐associated inhibitor LEKTI, a serine protease inhibitor.^9^ This study has found that dysfunction of* SPINK5* is mainly related to Netherton Syndrome (NS). NS is caused by loss of expression or dysfunction of LEKTI due to mutation of *SPINK5* gene.^10^ There were few studies exploring the relationship between *SPINK5* and human cancer. There was mRNA microarray analysis showed that *SPINK5* was downregulated in esophageal squamous cell carcinoma.^11^ However, the mechanism of action of SPINK5 in the development of esophageal cancer is still unclear.

In this study, we first explored the mechanism of action of *SPINK5* in the development of esophageal cancer. We found that *SPINK5 *acted as a tumor suppressor in esophageal cancer to inhibit proliferation, migration, and migration of esophageal cancer cells via inhibiting Wnt/β‐catenin signaling pathway, which provided an inspiration for exploring the mechanism of action of *SPINK5* in tumorigenesis and development, and provides a theoretical basis for the search for new therapeutic targets for esophageal cancer.

## 2 MATERIALS AND METHODS

### 2.1 Tissue sample and cell culture

A total of 2 esophageal tissue microarrays were used in this study. One tissue microarray containing 12 esophageal cancer tissues and their matched esophageal cancer tissues were purchased from Alenabio Company (Xi'an, China). The other tissue microarray contains 205 cases of esophageal cancer tissue which was from the tissue samples library of our laboratory. KYSE510, ECA109, and HEK293T cells were purchased from the China Center for Type Culture Collection (CCTCC; Chinese Academy of Sciences, Shanghai, China). KYSE510 cells were cultured in RPMI1640 medium (HyClone, USA) supplemented with 10% fetal bovine serum (Gibco). ECA109 and HEK293T cells were cultured in DMEM medium (HyClone, USA) supplemented with 10% fetal bovine serum (Gibco).

### 2.2 Plasmids, siRNAs, antibodies, and construction of stable cell line

The *SPINK5* overexpression plasmid was cloned into the pflag‐CMV vector by nested PCR using the CDS sequence of the *SPINK5* gene (NM_001127698.1). The primers of plasmid construction could be seen in Table S1. The TOP/FOP flash reporter plasmids containing wild‐type (TOP flash) or mutated (FOP flash) TCF/LEF DNA binding sites were conserved in our laboratory.

The siRNAs of *SPINK5 *were purchased from Genepharma Company (Shanghai, China) and contained 3 pairs of sequences, which were siRNA1 at nucleotides 1065 bp, siRNA2 at nucleotides 2046 bp, and siRNA3 at nucleotides 2910 bp. The sequences could be seen in Table S1. Transfection of plasmid and siRNA was carried out using Lipofectamine 2000 reagent (Invitrogen Co., Ltd.). The procedure of transfection was according to the instructions of Lipofectamine 2000 reagent. In the case of transfection of siRNA, siRNA1, siRNA2, and siRNA3 were mixed in equal proportions and then transfected into cells as the siRNA group.

The antibodies in this study were used for western blot and immunohistochemistry, including SPINK5 (Proteintech, USA), flag (Proteintech, USA), β‐catenin (Proteintech, USA), GSK3β (Proteintech, USA), c‐myc (Proteintech, USA), cyclin D1 (Proteintech, USA), β‐actin (Proteintech, USA), p‐β‐catenin (Cell Signaling Technology, USA), p‐GSK3β (Cell Signaling Technology, USA), and the goat anti‐rabbit IgG (Proteintech, USA) and goat anti‐mouse IgG (Proteintech, USA).

The lentivirus vectors LV5‐*SPINK5*, and LV5‐NC were purchased from Genepharma Company. For lentiviral stock preparation, HEK293T cells were co‐transfected with LV5‐SPINK5 and 3 packaging plasmids (pGag‐Pol, pRev, and pVSVG). Supernatants containing packaged lentivirus were collected after 48 hours, passed through a 0.45‐µm filter and added to ECA109 cells along with 1 µg/mL polybrene (Sigma). Infected cells were selected in puromycin‐containing medium.

### 2.3 Cell proliferation assay

The ability of cell proliferation was measured by CCK‐8 assay and plate colony formation assay. The procedure of the CCK8 assay was as follows. The cells in the logarithmic growth phase were seeded at a density of 1 × 10^3^ per well in 96‐well plates, and the proliferation of cells in each treatment group was detected at 0h, 24h, 48h using the CCK8 detection kit. For the plate colony formation assay, the cells in the logarithmic growth phase were inoculated into the 6‐well plate at a density of 200 per well. After further culture for 1 week, the cells were stained with crystal violet solution, and the growth of the cells in each treatment group was observed. Cell counting was performed by image J software.

### 2.4 Cell migration and invasion assay

Cell migration and invasion ability was measured by Transwell assay. For cell migration assay, cells in logarithmic growth phase were seeded at the upper transwell chamber insert (Corning, USA) at a density of 2 × 10^4^ cells per well. The chamber was placed in a 24‐well plate in which the upper chamber contained serum‐free cell culture medium and the lower chamber contained 10% FBS complete medium. The culture was continued for 24 hours. The medium was discarded, and stained with a crystal violet solution to observe the number of migrated cells. For cell invasion assay, Matrigel gel was uniformly applied to the upper chamber in advance, and then the cells were inoculated. The other procedures were the same as those in the cell migration experiment.

### 2.5 TOP/FOP flash assay

The TOP/FOP flash assay is a reporter gene assay used to detect Wnt/β‐catenin signaling pathway activity.^12^ The TOP flash reporter plasmid contains a wild‐type (CCTTTGATC) TCF/LEF binding site, and the FOP flash reporter plasmid contains a muted (CCTTTGGCC) TCF/LEF binding site. The cells in the logarithmic growth phase were seeded in 24‐well plates, and the target gene overexpression plasmid or siRNA was co‐transfected into the cells with TOP flash or FOP flash using Lipofectamine 2000 reagent. After 48 hours, the Dual Luciferase Reporter Assay Kit (Promega) was used to detect the reporter gene activity of each group of cells.

### 2.6 Western blot

The cell proteins were extracted from each group of cells by RIPA lysate and sonicator, and the concentration of each group of protein samples was detected by BCA protein concentration detection kit to prepare protein samples. An equal amount of protein sample (40 μg) was added to a 10% SDS‐PAGE gel and electrophoresed at 60 and 110 V, respectively. After the electrophoresis, the protein in the SDS‐PAGE gel was transferred to the PVDF membrane at a moderate current. The PVDF membrane was then blocked with 5% skim milk for 1 hour, and the corresponding protein antibody was separately incubated overnight according to the molecular weight of the protein of interest. Then, the PVDF membrane was separately incubated with the corresponding HRP‐labeled secondary antibody, and finally observed and photographed by the chemiluminescence imaging system (Tanon, Shanghai, China).

### 2.7 Immunohistochemistry

Immunohistochemistry was used to detect the expression level of the protein in esophageal tissue. The tissue chip was dewaxed, and after dehydration, the endogenous peroxidase in the tissue was inactivated using 1 mmol/L sodium citrate buffer (pH 6.0). After blocking with goat serum for 30 minutes, the antibodies of the protein of interest were separately incubated overnight at 4°C. The next day, the biotin‐labeled secondary antibody was incubated with the tissue chip for 1 hour at room temperature, followed by incubation with streptavidin‐labeled horseradish peroxidase for 1 hour and finally with 3, 3‐diaminobenzidine (DAB), and the depth of the color represents the expression level of the target protein. The Pannoramic MIDI automatic digital slide scanner (3DHISTECH Ltd., Budapest, HUNGARY) was used for image capture. Quantification of target protein staining was performed using IHC profiler in ImageJ.^13^

### 2.8 Nude mouse orthotopic transplantation tumor models

The tumorigenic ability of esophageal cancer cells ECA109 after *SPINK5* overexpression was determined by orthotopic transplanted tumor model in nude mice. A nude mouse model of orthotopic transplanted tumor was established by subcutaneous injection of ECA109 stable cell line (1 × 10^8^ cell/mL) in 4‐6 weeks old Balb/c nude mice (Beijing Vital River Laboratory Animal Technology Co., Ltd.). After the nude mice were sacrificed, the tumor weight and tumor volume were observed and recorded. All the animal protocols were approved by Zhang Zhongjing College of Traditional Chinese Medicine, Nanyang institute of Technology, China.

### 2.9 Statistical analysis

The data were presented as the mean ± standard deviation (*SD*). Each experiment was repeated at least 3 times. The variance analysis between groups was performed using a one‐way ANOVA. Chi squared‐tests were used to detect the significance of the relationship between expression of *SPINK5* and clinicopathologic features of esophageal cancer tissue microarray. *P < *0.05 was considered statistically significant.

## 3 RESULTS

### 3.1 SPINK5 expression is decreased in esophageal cancer tissues and is associated with clinicopathological features of esophageal cancer

**Figure 1:**
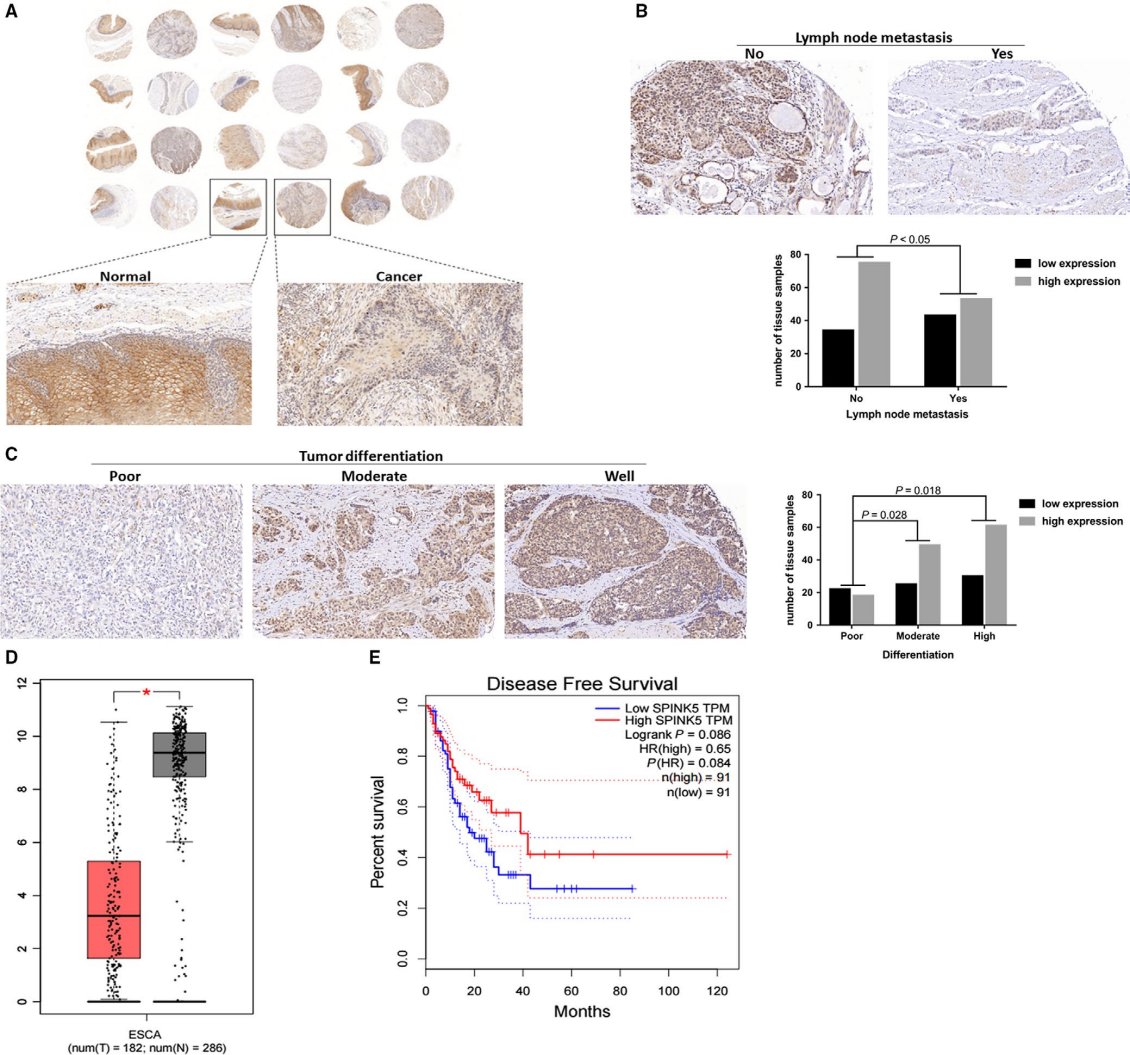
*SPINK5* is significantly downregulated in human esophageal cancer tissues. (A) Compared to normal esophageal tissues, SPINK5 protein expression was upregulated in esophageal cancer, which was detected in 12 cases of human esophageal cancer tissue microarray by immunohistochemistry. (B) The protein levels of SPINK5 in esophageal cancer tissues which occurs lymph node metastasis were lower than that in esophageal cancer tissues which have not lymph node metastasis. (C) The protein levels of SPINK5 in poor differentiation of esophageal cancer tissues were lower than that in moderate and well differentiation of esophageal cancer tissues. (D) The expression of *SPINK5 *was analyzed by bioinformatics in GEPIA database. (E) The relationship of *SPINK5* expression with disease free survival percent of esophageal cancer patient was analyzed by bioinformatics in GEPIA database

There are no studies to determine the difference in the expression levels of SPINK5 in normal esophageal and esophageal cancer tissues. Therefore, we detected the expression level of SPINK5 protein in 12 cases of esophageal cancer tissues and their matched normal esophageal tissues by immunohistochemistry. The results showed that the expression of SPINK5 protein was significantly reduced in 11 cases (11/12) of esophageal cancer, compared with normal esophageal tissues (Figure 1A). To further investigate the relationship between the expression level of SPINK5 protein and the clinicopathological features of esophageal cancer, we examined the expression level of SPINK5 protein in 205 cases of esophageal cancer by immunohistochemistry, the results showed that the expression level of SPINK5 protein was closely related to lymph node metastasis and pathological differentiation of esophageal cancer (Figure 1B,C, Table 1). When the expression level of SPINK5 protein was lower, the pathological differentiation of esophageal cancer patients was lower, and lymph node metastasis was more likely to occur. We analyzed the GEPIA database and found that the expression level of* SPINK5 *mRNA in esophageal cancer tissues was significantly lower than that in normal esophageal tissues (Figure 1D). In addition, although the expression level of *SPINK5* mRNA was not significantly different from the disease‐free survival rate of patients with esophageal cancer, however, based on the analysis results, we can find that the higher the expression level of *SPINK5* mRNA, the better the prognosis of patients with esophageal cancer (Figure 1E). These results showed that *SPINK5* was downregulated in esophageal cancer, and maybe related to the development of esophageal cancer.

**Table 1:** SPINK5 expression and clinicopathologic characteristics of esophageal cancer TMA

	Cases (n = 205)	SPINK5 protein expression in cancer tissue (n)		*P* value
		Low expression	High expression	
Age				
＜60	84	32	52	>0.05
≥60	121	45	76	
Gender				
Male	129	52	77	>0.05
Female	76	25	51	
T stage				
T1	31	12	19	>0.05
T2	46	17	29	
T3	95	35	60	
T4	33	13	20	
Lymph node metastasis				
No	109	34	75	**<0.05**
Yes	96	43	53	
Differentiation				
Well	91	30	61	**<0.02**
Moderate	74	25	49	
Poor	40	22	18	

*P* values were based on chi‐squared test.

Bold values were considered statistically significant.

### 3.2 SPINK5 significantly inhibits the proliferation, migration, and invasion of esophageal cancer cells

**Figure 2:**
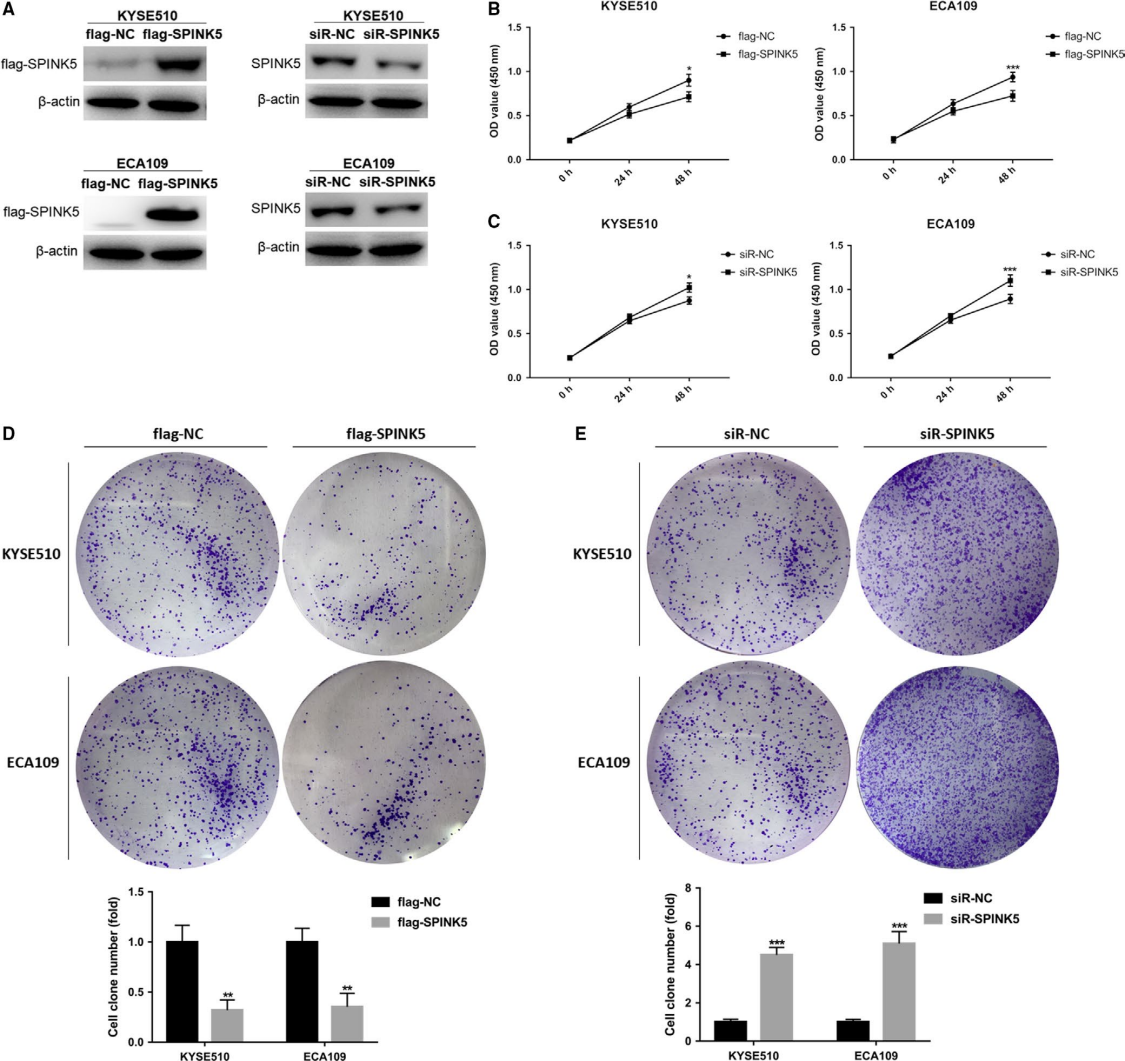
*SPINK5* promotes proliferation of esophageal cancer cells. (A) The cells were transfected with flag‐SPINK5 overexpression plasmid or flag‐NC negative control vector, and small interfering RNA of *SPINK5* (siR‐SPINK5) or negative control (siR‐NC) were detected by western blotting in KYSE510 and ECA109 cells. (B) *SPINK5* overexpression inhibits the proliferation of KYSE510 and ECA109 cells which was detected by the CCK‐8 assay. (C) *SPINK5* knockdown enhances the proliferation of KYSE510 and ECA109 cells which was detected by the CCK‐8 assay. (D) *SPINK5* overexpression inhibits the proliferation of KYSE510 and ECA109 cells which was detected by the colon formation assay. (E) *SPINK5* knockdown enhances the proliferation of KYSE510 and ECA109 cells which was detected by the colon formation assay. Values represent the mean ± *SD* from 3 independent measurements. *P < 0.05, **P < 0.01, ***P < 0.001

**Figure 3:**
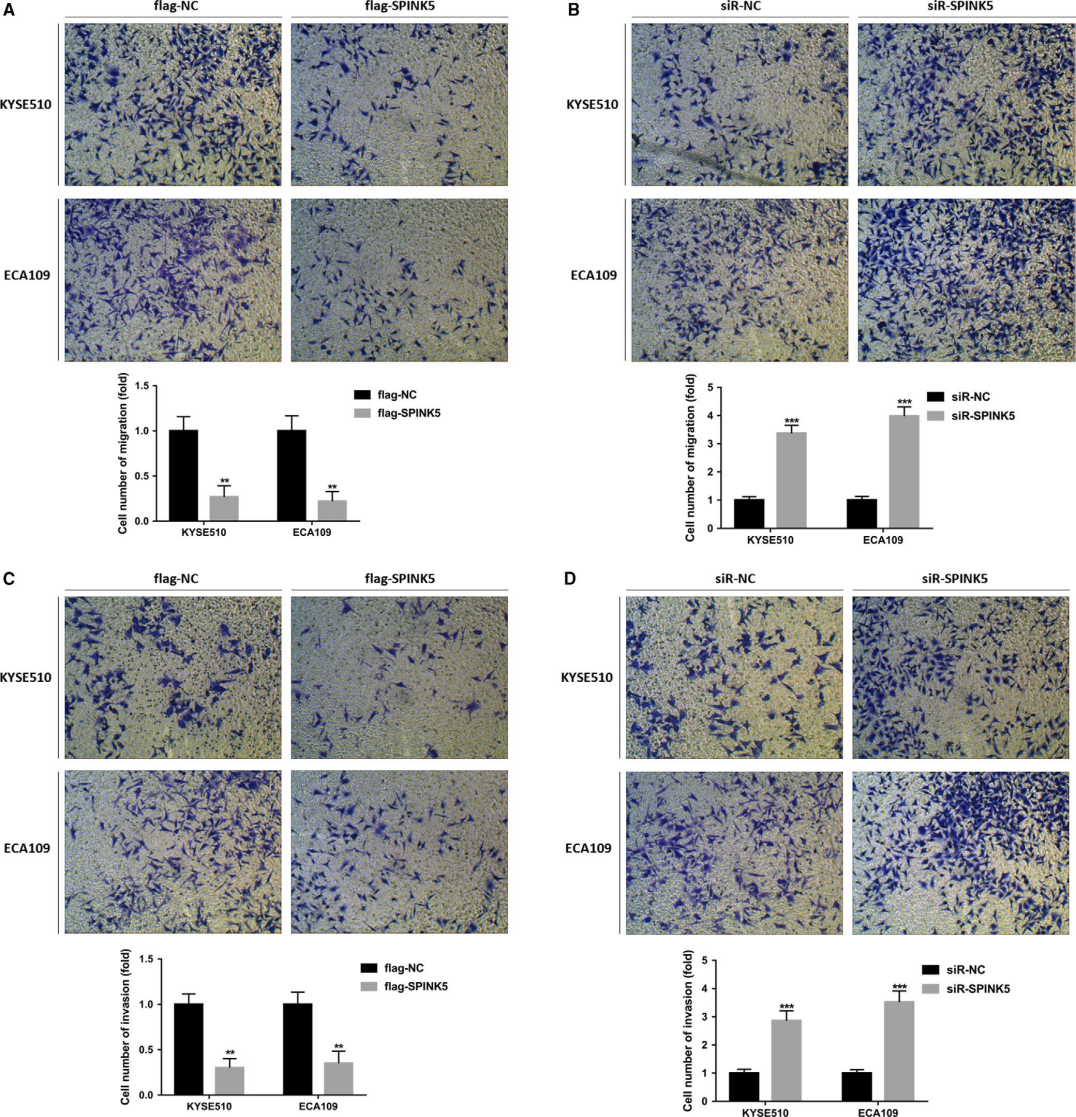
*SPINK5* significantly suppresses the migration and invasion of esophageal cancer cells. (A) *SPINK5* overexpression inhibits the migration of KYSE510 and ECA109 cells which was detected by Transwell assay. (B) *SPINK5* knockdown enhances the migration of KYSE510 and ECA109 cells which was detected by Transwell assay. (C) *SPINK5* overexpression inhibits the invasion of KYSE510 and ECA109 cells which was detected by Transwell assay. (D) *SPINK5* knockdown enhances the invasion of KYSE510 and ECA109 cells which was detected by Transwell assay. Values represent the mean ± *SD* from 3 independent measurements. **P < 0.01, ***P < 0.001

We have found that the expression level of *SPINK5* is significantly reduced during the development of esophageal cancer, so what role does *SPINK5* play in the development of esophageal cancer? We first constructed the overexpression plasmid and siRNA of *SPINK5* and then transfected into esophageal cancer cells KYSE510 and ECA109 (Figure 2A). Then, CCK‐8 assay and plate cloning assay were used to detect the effect of *SPINK5 *on the proliferation of esophageal cancer cells. The results showed that overexpression of *SPINK5* significantly inhibited the proliferation of esophageal cancer cells KYSE510 and ECA109 (Figure 2B,D), while knockdown of *SPINK5* significantly promoted proliferation of esophageal cancer cells KYSE510 and ECA109 (Figure 2C,E). In addition, metastasis is the leading cause of death in patients with esophageal cancer. We examined the effect of *SPINK5 *on the migration and invasion of esophageal cancer cells by Transwell assay. Transwell migration assay showed that overexpression of *SPINK5* significantly inhibited the migration of esophageal cancer cells KYSE510 and ECA109 (Figure 3A), while knockdown of *SPINK5* significantly promoted the migration of esophageal cancer cells KYSE510 and ECA109 (Figure 3B). Transwell invasion experiments were consistent with Transwell migration experiments (Figure 3C‐D). These results indicate that *SPINK5* acts as a tumor suppressor gene and participates in the development of esophageal cancer by inhibiting cell proliferation, migration, and invasion.

### 3.3 SPINK5 inhibits Wnt/β‐catenin signaling pathway in esophageal cancer

**Figure 4:**
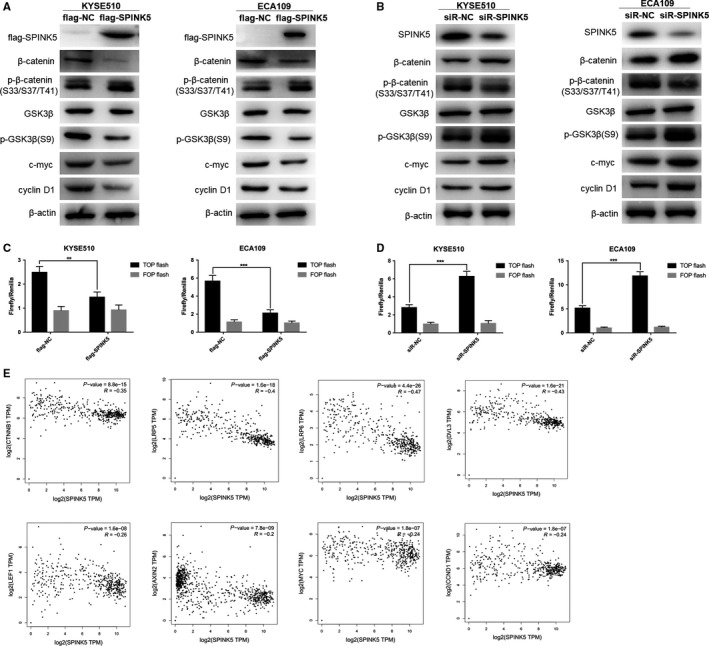
*SPINK5 *inhibits Wnt/β‐catenin signaling pathway in esophageal cancer cells. (A) The effect of transfecting with flag‐SPINK5 overexpression plasmid or negative control flag‐NC on the protein levels of flag‐SPINK5, β‐catenin, p‐β‐catenin (S33/S37/T41), GSK3β, p‐GSK3β(S9), c‐myc, cyclin D1 in KYSE510, and ECA109 cells. (B) The effect of transfecting with small interfering RNA of *SPINK5* (siR‐SPINK5) or negative control (siR‐NC) on the protein levels of flag‐SPINK5, β‐catenin, p‐β‐catenin (S33/S37/T41), GSK3β, p‐GSK3β(S9), c‐myc, cyclin D1 in KYSE510, and ECA109 cells. (C) *SPINK5* overexpression inhibits the activity of Wnt/β‐catenin signaling pathway which was detected by TOP/FOP flash assay in KYSE510 and ECA109 cells. (D) *SPINK5* knockdown increases the activity of Wnt/β‐catenin signaling pathway which was detected by TOP/FOP flash assay in KYSE510 and ECA109 cells. (E) The expression of *SPINK5* was negatively correlated with β‐catenin (CTNNB1), LRP5, LRP6, DVL3, LEF1, AXIN2, c‐myc (MYC), cyclin D1 (CCND1) expression which was analyzed in GEPIA database by bioinformatics methods. Values represent the mean ± *SD* from 3 independent measurements. **P < 0.01, ***P < 0.001

In the development of esophageal cancer, a variety of signaling pathways are involved, among which Wnt/β‐catenin signaling pathway is one of the most classical oncogenic signaling pathways.^14^ Its overactivation can promote the expression of target gene c‐myc and cyclin D1 which in turn to promote the proliferation, migration, and invasion of tumor cells. In esophageal cancer cells KYSE510 and ECA109, we transfected *SPINK5* overexpression plasmid and siRNA, respectively, and then detected the expression levels of key factors of Wnt/β‐catenin signaling pathway by western blot. The results showed that overexpression of *SPINK5* inhibited the protein expression levels of β‐catenin, p‐GSK3β(S9), c‐myc, and cyclin D1 and upregulated p‐β‐catenin (S33/37) in esophageal cancer cells KYSE510 and ECA109 (Figure 4A). Knockdown of *SPINK5* significantly inhibited the protein expression level of p‐β‐catenin (S33/37) and upregulated the protein expression levels of β‐catenin, p‐GSK3β (S9), c‐myc and cyclin D1 (Figure 4B). The TOP/FOP flash assay was used to detect Wnt/β‐catenin signaling pathway activity. In esophageal cancer cells KYSE510 and ECA109, overexpression of SPINK5 significantly inhibited TOP flash reporter activity (Figure 4C), while knockdown of* SPINK5* significantly increased TOP flash reporter activity (Figure 4D), suggesting that overexpression of *SPINK5 *inhibits Wnt/β‐catenin signaling pathway activity, while *SPINK5* knockdown is able to activate Wnt/β‐catenin signaling pathway. To further validate the association of *SPINK5* with Wnt/β‐catenin signaling pathways in esophageal cancer, we used bioinformatics to analyze the correlation between the expression levels of *SPINK5 *and Wnt/β‐catenin signaling pathways. The results showed a significant correlation between the expression levels of *SPINK5* and β‐catenin (CTNNB1), LRP5, LRP6, DVL3, LEF1, AXIN2, MYC, and cyclin D1 (CCND1) in esophageal cancer (Figure 4E). These results indicate that *SPINK5* is able to inhibit the Wnt/β‐catenin signaling pathway in esophageal cancer.

### 3.4 SPINK5 can affect the degradation of β‐catenin through GSK3β pathway in esophageal cancer cell

**Figure 5:**
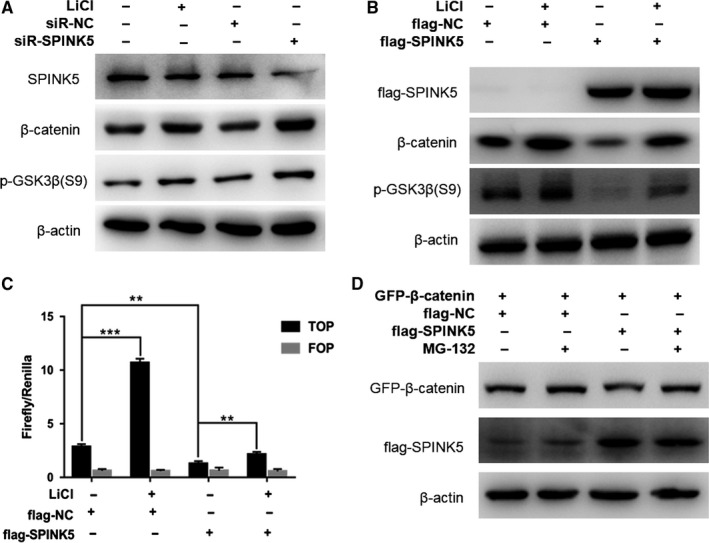
*SPINK5* promotes the degradation of β‐catenin via GSK3β pathway. (A) KYSE510 cells were transfected with flag‐NC, flag‐SPINK5, or treated with Licl for 48 h and harvested for western blot assay. (B) LiCl reverses inhibition of β‐catenin expression by *SPINK5* overexpression. flag‐SPINK5 or flag‐NC was transfected into ECA109 cells. Thirty‐six hours after transfection, the cells were treated with 30 mmol/L LiCl for 12 h and then harvested for western blotting analysis to detect the expression of β‐catenin, p‐GSK3β (S9) and flag‐SPINK5. (C) Flag‐SPINK5 or flag‐NC was transfected into ECA109 cells. Thirty‐six hours after transfection, the cells were treated with 30 mmol/L LiCl for 12 h and harvested for luciferase activity assay. (D) Proteasomes inhibitor MG132 reverses the effect of SPINK5 overexpression on β‐catenin degradation. ECA109 cells were cotransfected with pEGFP‐β‐catenin and flag‐SPINK5 or flag‐NC. Forty‐four hours after transfection, the cells were treated with 30μM MG132. Cells were harvested 4 h later to detect the expression of GFP‐β‐catenin and flag‐SPINK5 by western blotting. Values represent the mean ± *SD* from 3 independent measurements. **P < 0.01, ***P < 0.001

The cytoplasmic CK1, AXIN, APC, GSK3β, and other proteins form a degradation complex, which promotes the phosphorylation of the ser33 and ser37 of β‐catenin by binding to β‐catenin protein, which leads to the ubiquitination degradation of β‐catenin.^15^ In previous studies, we found that overexpression of *SPINK5* in esophageal cancer cells inhibited the expression of p‐GSK3β (S9), whereas knockdown of *SPINK5* upregulated the expression of p‐GSK3β (S9), suggesting that *SPINK5* can affect the activity of GSK3β. LiCl is often used as an inhibitor of GSK3β which promotes GSK3β phosphorylation and inactivation.^16^ We found that knockdown of *SPINK5* in esophageal cancer cells has a similar effect to LiCl and upregulates the expression level of p‐GSK3β (S9) (Figure 5A). Overexpression of *SPINK5* significantly reversed the upregulation of β‐catenin and p‐GSK3β (S9) protein expression by LiCl treatment (Figure 5B). TOP/FOP flash assay showed that overexpression of *SPINK5* significantly reversed the activation of Wnt/β‐catenin signaling pathway by LiCl treatment (Figure 5C). In esophageal cancer cells, we overexpressed β‐catenin (GFP‐β‐catenin) in combination with protease inhibitor MG132, the results showed that MG132 significantly upregulated the expression of GFP‐β‐catenin and reversed the overexpression of *SPINK5* to inhibit GFP‐β‐catenin (Figure 5D). These results suggest that SPINK5 can promote β‐catenin protein degradation by inhibiting GSK3β phosphorylation in esophageal cancer cells.

### 3.5 SPINK5 inhibits the proliferation and migration of esophageal cancer cells through Wnt/β‐catenin signaling pathway and inhibits the growth of esophageal cancer cells in nude mice

**Figure 6:**
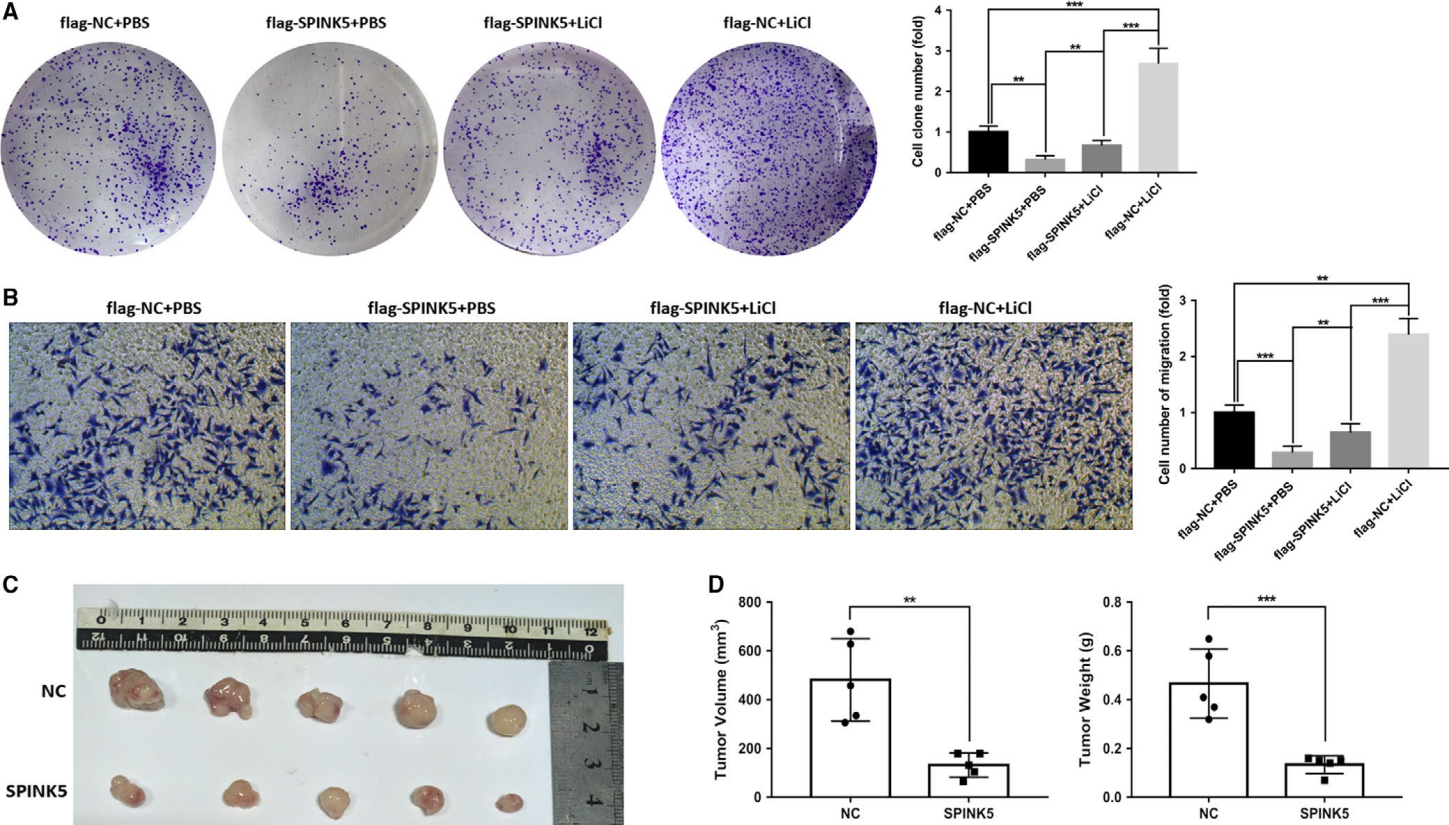
*SPINK5 *suppresses proliferation and migration of esophageal cancer cells via Wnt/β‐catenin signaling pathway. (A) GSK3β inhibitor LiCl (30mM) reverses the effect of *SPINK5 *overexpression on suppressing the proliferation of ECA109 cells. (B) GSK3β inhibitor LiCl (30 mmol/L) reverses the effect of* SPINK5* overexpression on suppressing the migration of ECA109 cells. (C) Compared to control group (NC), *SPINK5 *overexpression (*SPINK5*) significantly suppresses the xenografts growth in nude mouse model. (D) The volume and weight of xenografts in *SPINK5 *group and NC group were counted and analyzed. Values represent the mean ± *SD* from 3 independent measurements. **P < 0.01, ***P < 0.001

In esophageal cancer cells, we have determined that *SPINK5* regulates the Wnt/β‐catenin signaling pathway. Furthermore, we verified whether *SPINK5 *affects the proliferation and migration of esophageal cancer cells via the Wnt/β‐catenin signaling pathway. We used LiCl to activate the Wnt/β‐catenin signaling pathway, and then observed the effect of overexpression of *SPINK5 *on the proliferation and migration of esophageal cancer cells by plate colony formation assay and Transwell assay. The results showed that the proliferation and migration ability of esophageal cancer cells increased significantly after treatment with 30 mmol/L LiCl, while overexpression of *SPINK5* significantly inhibited the proliferation and migration of esophageal cancer cells, and reversed the promotion of LiCl treatment on the proliferation and migration of esophageal cancer cells (Figure 6A‐B). These results indicate that *SPINK5* can inhibit the proliferation and migration of esophageal cancer cells through the Wnt/β‐catenin signaling pathway. In addition, we further verified the effect of *SPINK5* on the growth of esophageal cancer cells in nude mice. We constructed a *SPINK5* overexpressing ECA109 esophageal cancer cell line, which was then implanted subcutaneously in nude mice. The results showed that overexpression of* SPINK5* significantly inhibited the growth of esophageal cancer cells in nude mice (Figure 6C‐D). These results indicate that *SPINK5* significantly inhibits the proliferation and migration of esophageal cancer cells and has potential as a therapeutic target for esophageal cancer.

## 4 DISCUSSION

The role of *SPINK* family genes in tumorigenesis and development is still unclear. Studies have shown that *SPINK1* is highly expressed in prostate cancer,^17^ colorectal cancer,^18^ liver cancer,^19^ and other tumor tissues,^20^ and promotes tumors occurrence and development by activating PI3K/AKT, MAPK/ERK, and other signaling pathways.^21^ *SPINK7* is elevated in normal adult esophageal tissue, but decreased or lost in esophageal and paracancerous tissues. *SPINK7* is downregulated in esophageal cancer, and inhibits invasion, and migration of tumor cells through uPA/plasmin, uPAR/FPRL1, and other signaling pathways.^22^ However, the relationship between *SPINK5* and tumors is not clear. In this study, we found that the protein expression level of *SPINK5* was significantly reduced in esophageal cancer tissues relative to normal esophageal tissues, which was consistent with the results of previous mRNA microarray analysis.^11^ In addition, in vitro studies showed that *SPINK5* overexpression significantly inhibited the proliferation, migration, and invasion of esophageal cancer cells. These results suggest that *SPINK5* may be involved in the development of esophageal cancer as a tumor suppressor gene.

The occurrence and development of malignant tumors, such as esophageal cancer, are closely related to downregulation or inactivation of tumor suppressor genes, upregulation or activation of oncogene expression, activation of oncogenic signaling pathways, and inactivation of tumor suppressor signaling pathways.^23^ The Wnt/β‐catenin signaling pathway is one of the most classical oncogenic signaling pathways. Excessive activation of the Wnt/β‐catenin signaling pathway is often accompanied by the development of malignant tumors such as esophageal cancer.^24^ Although the Wnt/β‐catenin signaling pathway is essential for embryonic development, cell division and other physiological processes, however, when the Wnt/β‐catenin signaling pathway is over‐activated, the expression of target genes such as c‐myc and cyclin D1, which are related to cell proliferation and migration, is upregulated, which promotes the cell proliferation and migration, leading to malignant transformation of cells.^25^ Therefore, exploring the upstream and downstream regulatory mechanisms of Wnt/β‐catenin signaling pathway is a hot spot in current cancer research. In this study, we found through bioinformatics analysis that the expression level of *SPINK5* is closely related to Wnt/β‐catenin signaling pathway in esophageal cancer. The results of the In vitro study further demonstrated that *SPINK5* significantly inhibits the activity of the Wnt/β‐catenin signaling pathway in esophageal cancer cells.

The Wnt/β‐catenin signaling pathway is a complex and precise signaling pathway in which the localization and expression level of β‐catenin protein is a key point. When the Wnt/β‐catenin signaling pathway is inactivated, CK1, AXIN, APC, GSK3β, and other proteins in the cytoplasm form a degradation complex, which promotes the ser33 and ser37 phosphorylation of β‐catenin by binding to β‐catenin protein, leading to the degradation of β‐catenin by ubiquitination.^26^ When the Wnt/β‐catenin signaling pathway is activated, the Wnt family protein binds to the receptor Frizzle protein, as well as the co‐receptors LRP5 and LRP6 on the cell membrane, and transmit an activation signal to the cytoplasm through the DVL protein, leading to the dissociation of degradation complexes in the cytoplasm, the degradation of β‐catenin protein is inhibited, and the β‐catenin protein in the cytoplasm is aggregated, which then enters the nucleus and binds to the TCF/LEF transcriptional regulatory complex to activate the transcriptional expression of the target gene.^27^ Therefore, the expression level and localization of β‐catenin protein in cells determines the activity of the Wnt/β‐catenin signaling pathway. In this study, we found that *SPINK5* overexpression significantly inhibited the expression of β‐catenin, while knockdown of *SPINK5 *promoted the expression of β‐catenin. GSK3β is one of the key factors in the degradation complex, which can promote the phosphorylation of serine at positions 33 and 37 of β‐catenin protein. The kinase activity of GSK3β is closely related to the phosphorylation of ser9.^28^ GSK3β is inactivated by phosphorylating at Ser9, so the expression level of p‐GSK3β (S9) in cells is one of the indicators to measure the activity of degradation complex.^29^ In this study, we found that *SPINK5* inhibits the expression level of p‐GSK3β (S9), but has no significant effect on the expression level of GSK3β. Furthermore, using the GSK3β inhibitor LiCl in combination with *SPINK5* overexpression or knockdown, we further determined that *SPINK5 *can activate GSK3β and inhibit the activity of Wnt/β‐catenin signaling pathway by affecting the GSK3β/β‐catenin degradation complex.

In the process of tumor development, activation of Wnt/β‐catenin signaling pathway can upregulate cell proliferation and migration‐related target genes, such as c‐myc and cyclin D1, which promote cell proliferation and migration.^30^ Therefore, we further demonstrated that *SPINK5* overexpression could reverse the effect of LiCl treatment, which confirmed that *SPINK5 *could indeed inhibit the proliferation and migration of esophageal cancer cells by inhibiting the activity of Wnt/β‐catenin signaling pathway. In addition, the inhibitory effect of *SPINK5* overexpression on the growth of esophageal cancer cells was further verified in nude mice, suggesting that *SPINK5* may be a new therapeutic target for esophageal cancer.

In conclusion, in this study, we first explored the role of *SPINK5 *as a tumor suppressor gene in the development of esophageal carcinoma, and further found that *SPINK5* can inhibit the Wnt/β‐catenin signaling pathway to play a role in the proliferation, migration, and invasion of esophageal cancer cells. In addition, in this study, we also found that *SPINK5* is mainly expressed on the cell membrane in normal esophageal tissues, while SPINK5 is mainly in the cytoplasm and nucleus in esophageal cancer tissues. In general, the position of protein determines its function. Therefore, the correlation between the transposition of SPINK5 and its function is one of the directions of future research.

## CONFLICT OF INTEREST

None declared.

## Supporting information

TableS1
