# Supplementary material for: A novel tumor suppressor SPINK5 targets Wnt/β‐catenin signaling pathway in esophageal cancer
Source: Cancer Med. 2019 Mar 13;8(5):2360–71. doi: 10.1002/cam4.2078 (PMC6537088; doi:10.1002/cam4.2078)
Supplement: Supplementary file 1 — TableS1 [file CAM4-8-2360-s001.docx]

| **primers or siRNAs** | **sequences** |
| --- | --- |
| pflag-SPINK5 | Forward 1: 5'-GCGACTTGCATCGTCTTC-3'  Reverse 1: 5'-ATGGCTTTCAACAATCTTCC-3'  Forward 2: 5'-GACGATGACGACAAGCTTAAGATAGCCACAGTGTCAGTG-3'  Reverse 2: 5'-GGATGCCACCCGGGATCCTTAATAAATAACCTTTGCATT-3' |
| SPINK5-siRNA1 | sense: 5'- CCAUGUGUCAAGCCUACUUTT-3'  anti-sense: 5'- AAGUAGGCUUGACACAUGGTT-3' |
| SPINK5-siRNA2 | sense:5'- GCAAGACCCAUGGCAACAATT-3'  anti-sense: 5'- UUGUUGCCAUGGGUCUUGCTT-3' |
| SPINK5-siRNA3 | sense: 5'- GCAGUGAAUUUCGAAACUATT-3'  anti-sense: 5'- UAGUUCGAAAUUCACUGCTT-3' |
| negative control siRNA | sense: 5'- UUCUCCGAACGUGUCACGUTT-3'  anti-sense: 5'- ACGUGACACGUUCGGAGAATT-3' |

**Table 1. The sequences of primers and siRNAs**
